# *SHISA3* Promoter Methylation Is a Potential Diagnostic and Prognostic Biomarker for Laryngeal Squamous Cell Carcinoma

**DOI:** 10.1155/2017/9058749

**Published:** 2017-02-16

**Authors:** Zhisen Shen, Chongchang Zhou, Jinyun Li, Dong Ye, Hongxia Deng, Bin Cao, Wenjuan Hao, Lexi Lin, Yuna Zhang

**Affiliations:** ^1^Department of Otorhinolaryngology Head and Neck Surgery, Lihuili Hospital, Ningbo University, Ningbo, Zhejiang 315040, China; ^2^School of Medicine, Ningbo University, Ningbo, Zhejiang 315211, China

## Abstract

The purpose of this study was to evaluate the contribution of* SHISA3* promoter methylation to laryngeal squamous cell carcinoma (LSCC).* SHISA3* promoter methylation status and expression were determined using methylation-specific polymerase chain reaction (MSP) and quantitative real-time PCR (qRT-PCR) in 93 paired LSCC and adjacent normal tissues, respectively. Furthermore, the regulatory function of the* SHISA3 *promoter fragment was analyzed using a luciferase reporter assay. The results reveal that there is a significant increase in* SHISA3 *methylation in LSCC tissues compared with corresponding nontumor tissues (*P* = 4.58*E* − 12). The qRT-PCR results show a significant association between* SHISA3* methylation and expression in LSCC (*P* = 1.67*E* − 03). In addition, the area under the receiver operating characteristic curve was 0.91. Consequently, a log-rank test and multivariate Cox analysis suggest that* SHISA3* promoter hypermethylation is a predictor of poor overall survival for LSCC (log-rank* P* = 0.024; HR = 2.71; 95% CI = 1.024–7.177;* P* = 0.047). The results indicate that* SHISA3* promoter hypermethylation might increase the risk of LSCC through regulation of gene expression and is a potential diagnostic and prognostic biomarker for LSCC.

## 1. Introduction

Laryngeal cancer is one of the most common malignant tumors of the head and neck, and the major pathological type is squamous cell carcinoma [1]. Despite great progress in the prevention, diagnosis, and treatment of laryngeal squamous cell carcinoma (LSCC) in recent decades, the global incidence of LSCC remains high and the LSCC survival rate, a key measure of the effectiveness of therapeutic interventions, is still unsatisfactory, especially in Southeast Asia and Eastern Europe [2–4]. Currently, the main diagnostic methods for LSCC are histopathological examination through laryngoscopy and assisted diagnostic systems, including computed tomography and magnetic resonance imaging. However, because of nonspecific symptoms in early stage LSCC, a low rate of early diagnosis makes treatment challenging. It has been reported that LSCC patients' survival rates decrease dramatically when the tumor is diagnosed at an advanced stage [5]. Thus, identification of effective early biomarkers for LSCC is critical to improving patient outcomes.

The pathological mechanism of LSCC is complicated and involves genetic, epigenetic, and environmental factors [6]. DNA methylation, an important epigenetic modification [7], is considered as a hallmark of cancer and is significantly related with various malignancies, including LSCC [8–10]. DNA methylation is precisely regulated by DNA methyltransferases [11]. 5-Methylcytosine mainly occurs among the cytosine-phosphate-guanine (CpG) dinucleotides in regions of densely clustered CpGs, known as CpG islands [11]. The majority of CpG islands are observed in the neighborhood of gene promoters and are often devoid of methylation [12]. Aberrant hypermethylated cytosines among CpG dinucleotides have been proven to be associated with the transcriptional inactivation of genes [12]. Furthermore, aberrant DNA methylation has been shown to play a role in the diagnosis and prognosis of a wide range of cancers, including LSCC [13, 14]. Therefore, the identification of DNA methylation biomarkers specific for LSCC could greatly enhance the power of diagnosis and prognosis of LSCC.

The human shisa family member 3* (SHISA3)* is located on chromosome 4p13 and was recently discovered to be a tumor suppressor gene, which suppresses the tumorigenesis, invasion, and metastasis of lung cancer through the degradation of *β*-catenin of the Wnt signaling pathway [15]. Recent reports have demonstrated that hypermethylation of the* SHISA3 *promoter region is a common event in colorectal cancer tissues and cell lines, and can serve as an independent predictor of poor overall survival for colorectal cancer patients [16]. However, the association between* SHISA3* promoter methylation and LSCC has not yet been fully investigated.

In the present study, the aim was to explore the contribution of* SHISA3* promoter methylation to LSCC pathogenesis and its potential diagnostic and prognostic value for LSCC.

## 2. Materials and Methods

### 2.1. Patient Characteristics and Tissue Specimen Collection

A total of 93 LSCC patients, who underwent surgery at the Department of Otolaryngology, Head and Neck Surgery, in Ningbo Lihuili Hospital between June 2010 and April 2015, were recruited to the current study. None of the patients underwent chemotherapy or radiotherapy before surgery. All patients have signed the informed consent for the surgical procedure and tissue collection. All of the specimens were collected at the time of surgery and immediately stored in liquid nitrogen at −80°C after excision. In addition, tumor tissues and their paired adjacent tissues were subjected to histological diagnosis by two pathologists according to the World Health Organization classification. There were 45 well-differentiated cases, 35 moderately differentiated cases, and 13 poorly differentiated cases. The clinical stages were confirmed according to the TNM staging system of the AJCC 7th edition (2010). There were 29 Stage I cases, 19 Stage II cases, 12 Stage III cases, and 33 Stage IV cases. Patients were followed up for 60 months. Median follow-up was 41 months, with an interquartile range of 32–52 months. During follow-up, five patients were lost and 34 patients died. All the experiments were approved by the Ethical Committee of Ningbo Lihuili Hospital.

### 2.2. DNA Extraction and Bisulfite Modification

Genomic DNA was isolated from 93 paired samples using the QIAamp DNA Mini Kit (Qiagen, Hilden, Germany) according to the manufacturer's protocol. Then, purified DNA was subjected to bisulfite modification using the ZYMO EZ DNA Methylation-Gold Kit, following the manufacturer's instruction (Zymo Research, Orange, CA, USA).

### 2.3. Methylation-Specific Polymerase Chain Reaction (MSP) Assay

The methylation status of the* SHISA3* promoter region was tested using the MSP assay. The primer sequences are shown in Table 1. Each MSP amplification was performed using 2 *μ*L of bisulfite-modified DNA templates in a 20 *μ*L reaction volume containing 1 *μ*M each of forward and reverse primers, 0.2 mM dNTPs, 10x PCR buffer, and 2.5 U of Hot Start DNA Polymerase (Qiagen, Hilden, Germany). The polymerase chain reaction (PCR) conditions were as follows: denaturation at 95°C for 10 min, followed by 35 cycles of 93°C for 30 s, 56°C for 40 s and 72°C for 50 s, and a final extension at 72°C for 10 min. A water blank was used as a negative control. The generated PCR products were subjected to electrophoresis at 100 V for 30 min, using 2% agarose gel stained with ethidium bromide and visualized under UV illumination. Samples were considered as methylated when there was a clearly visible band (204 bp) on the gel, when using the methylated primers. PCR products were also sequenced to verify the MSP results.

### 2.4. Total RNA Extraction and Quantitative Real-Time PCR Assay

Total RNA was extracted from 35 paired LSCC and normal tissues using TRIzol reagent (Invitrogen, Carlsbad, CA, USA) following the manufacturer's protocol. The detailed procedure for the quantitative real-time PCR (qRT-PCR) experiment has been reported previously [17]. The PCR conditions were as follows: 95°C for 10 min and then 45 amplification cycles of 94°C for 20 s, 55°C for 30 s, and 70°C for 30 s. The cycle threshold (Ct) values were recorded for both* SHISA3* and* GAPDH*, which was used as an endogenous control. The expression level of* SHISA3* was calculated using the ΔCt method. All the samples were assayed in triplicate. The primer sequences for the qRT-PCR are listed in Table 1.

### 2.5. Cell Culture

Human HEK293T cells were purchased from the cell bank of the Chinese Academy of Sciences (Shanghai, China). The 293T cells were cultured in Dulbecco's modified Eagle's medium (DMEM; HyClone, Logan, Utah) supplemented with 10% fetal bovine serum (FBS) (ExCell Biology, Shanghai, China), 100 U/mL penicillin, and 100 *µ*g/mL streptomycin. The incubators were maintained at 37°C in a 5% CO_2_ atmosphere.

### 2.6. Construction of Recombinant Plasmids

A fragment of the* SHISA3* promoter (−1479 to −276) was amplified, which included the sequence analyzed by MSP. The primer sequences were 5′-CTTACGCGTGCTAGCCCTGTCCTAAGAAATATGTAACTCTAAGAGAG-3′ for the forward primer and 5′-CGCAGATCTCGAGCCCGCTCATAGCGCTCCCCGC-3′ for the reverse primer. The recombinant plasmid (pGL3-*SHISA3*) concatenated the target fragment of* SHISA3* and the pGL3 Basic vector (Promega, Madison city, WI, USA) via a DNA Ligation Kit (TaKaRa, Japan). The pRL-SV40 vector (Promega, Madison city, WI, USA) with the* Renilla* luciferase gene was used as an internal control in this study.

### 2.7. Transfection and Reporter Gene Activity Assay

The 293T cells in the exponential growth phase were plated on 24-well plates in DMEM with 10% FBS. After 12 h, cells of 70% attachment were transfected with pGL3-*SHISA3* and pRL-SV40 according to the manufacturer's protocol (TransLipid HL Transfection Reagent, TransGen Biotech, Beijing, China). After 4–6 h, the medium was exchanged for fresh DMEM with 10% FBS. After 18–72 h,* Renilla* and firefly luciferase activity were measured following the manufacturer's protocol (Dual-Luciferase® Reporter Assay Systems, Promega, Madison city, WI, USA).

### 2.8. Statistical Analyses

All the statistical analysis was performed using SPSS v18.0 (SPSS Inc., Chicago, IL, USA). A chi-squared test or Fisher's exact test was performed to evaluate the difference in* SHISA3* promoter methylation frequency between different groups. The comparison of the expression level was analyzed using paired Student's* t*-tests. The diagnostic value of* SHISA3* was assessed using the receiver operating characteristic (ROC) curve and the area under the curve (AUC) [18]. The overall survival (OS) curves were calculated using Kaplan–Meier analysis. Log-rank tests and multivariate Cox proportional hazard models were used to test the prognostic value of* SHISA3* methylation for LSCC patients. A two-tailed* P* value of less than 0.05 was considered statistically significant. All the figures were drawn using the GraphPad Prism 6 software (GraphPad, San Diego, CA).

## 3. Results

### 3.1. Methylation Status of* SHISA3* in LSCC and Paired Normal Tissues

**Figure 1:**
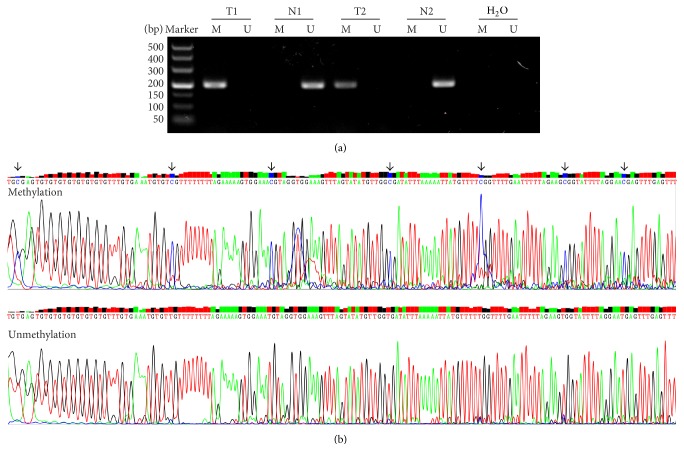
Agarose gel image and sequencing results of* SHISA3*. (a) Representative methylation-specific polymerase chain reaction (MSP) results regarding* SHISA3* in two paired LSCC and normal tissues. Water was used as a negative control. The methylated CpG sites were highlighted by arrow. T: tumor; N: adjacent normal tissue; M: methylation; U: unmethylation. (b) The sequencing results of methylation and unmethylation PCR products.

*SHISA3* methylation status in 93 LSCC tissue samples and adjacent normal tissue samples was tested using the MSP assay. The representative agarose gel electrophoresis images and the sequencing results are shown in Figure 1. This study reveals that the methylation frequency of the* SHISA3 *promoter is significantly greater in LSCC tissues than in corresponding normal tissues (*P* = 4.58*E* − 12). The* SHISA3* promoter was methylated in 66 of 93 (71%) LSCC tissue samples and only in 19 of 93 (20%) adjacent normal tissue samples. Of those patients with methylated* SHISA3* in adjacent normal tissues, methylated* SHISA3* was also observed in their paired LSCC tissues.

### 3.2. Association between* SHISA3* Promoter Methylation and Expression

**Figure 2:**
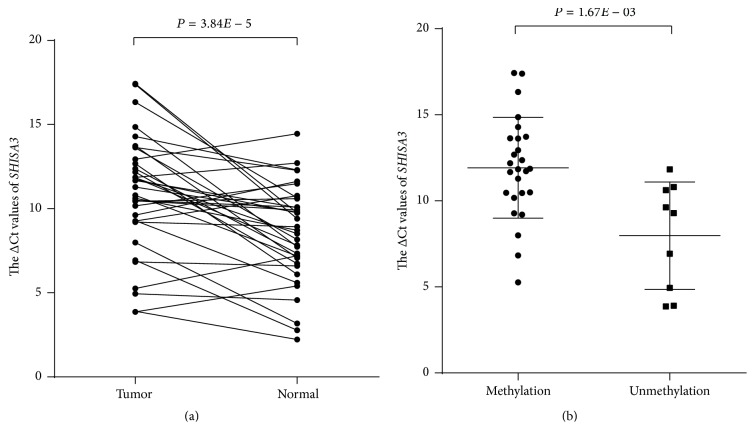
The expression of* SHISA3* assessed using quantitative RT-PCR assay. (a) Expression levels of* SHISA3* in LSCC and adjacent normal samples (*n* = 35). The* SHISA3* expression level was significantly lower in tumor tissues than in corresponding normal tissues (*P* = 3.84*E* − 05). (b) Expression levels of* SHISA3* in the methylation group (*n* = 26) and unmethylation group (*n* = 9). The* SHISA3* expression levels were significantly lower in the methylation group compared with the unmethylation group (*P* = 1.67*E* − 03).

Using qRT-PCR in 35 paired LSCC samples, it was found that the expression of* SHISA3* in LSCC tissues was significantly downregulated compared with adjacent normal tissues (*P* = 3.84*E* − 05, Figure 2(a)).* SHISA3* promoter methylation was observed in 74% (26/35) of these patients. In addition, the analysis showed that the expression of* SHISA3* was significantly lower in the methylated group compared with the unmethylated group (*P* = 1.67*E* − 03, Figure 2(b)).

### 3.3. Reporter Gene Activity Assay

**Figure 3:**
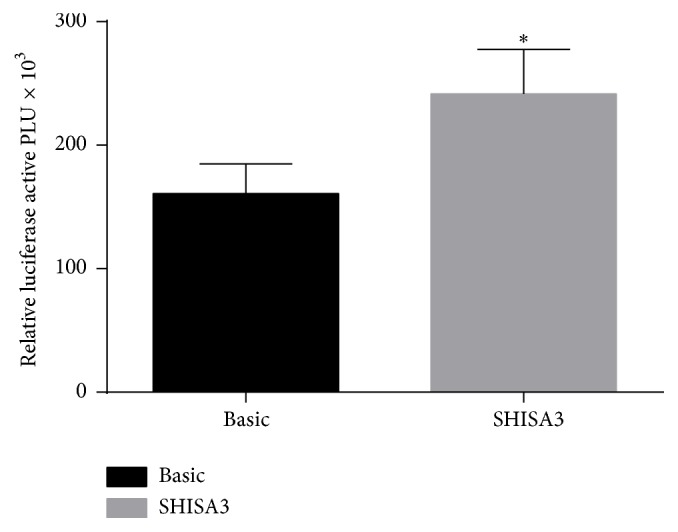
Activity analysis of fragment of promoter region of* SHISA3* gene. Asterisk symbol indicates* P* < 0.05 (*∗*).

In order to verify the transcriptional activity of the tested* SHISA3* promoter region, an in vitro luciferase reporter assay was performed. The results show that the pGL3*-SHISA3* plasmid, which contains a fragment of the* SHISA3* promoter region, had a significantly higher luciferase activity (*P* < 0.05, Figure 3) and imply that the hypermethylation of the target fragment of* SHISA3* may be responsible for its downregulation.

### 3.4. Association between* SHISA3* Promoter Methylation Status and Clinicopathological Parameters

The association between* SHISA3* promoter methylation status and the clinicopathological parameters of the LSCC patients, including age, gender, smoking behavior, histological grade, clinical stage, tumor stage, and lymph node metastasis, was assessed (Table 2). The results show that* SHISA3* promoter methylation is significantly associated with histological grade (*P* = 0.02), clinical stage (*P* = 0.02), tumor stage (*P* = 0.05), and lymph node metastasis (*P* = 0.03). However, no significant correlation between* SHISA3* promoter methylation and any other parameters was observed.

### 3.5. The Diagnostic and Prognostic Value of* SHISA3* Promoter Methylation for LSCC

**Figure 4:**
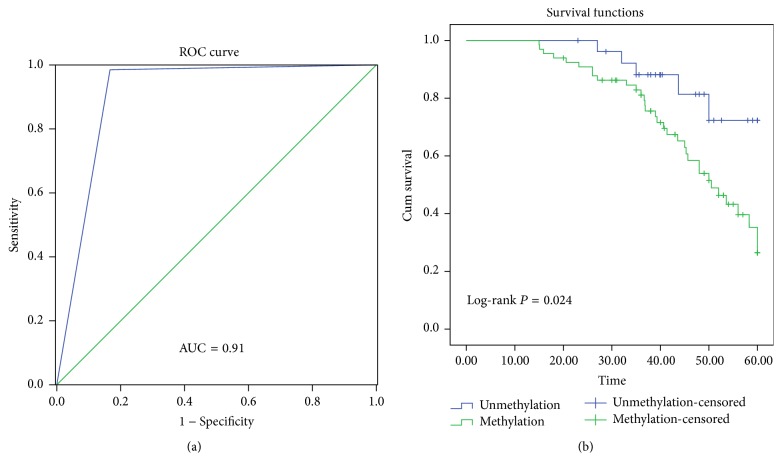
The receiver operating characteristic (ROC) curve and survival curve. (a) The ROC analyses of the curve. The area under the curve was 0.91. (b) The survival curve of patient groups according to* SHISA3* methylation status. The* SHISA3* methylation group showed significantly worse survival rates than the* SHISA3* unmethylation group (log-rank* P* = 0.024).

A ROC curve was plotted to evaluate the diagnostic value of* SHISA3* promoter methylation (Figure 4(a)). The area under the ROC curve is 0.91 (*P* < 0.01), while the sensitivity and specificity with the maximum Youden index are 0.99 and 0.83, respectively. In the current study, the prognostic value of* SHISA3* methylation status in LSCC was also investigated. As shown in Figure 4(b), the survival curves demonstrate that hypermethylated* SHISA3* is significantly associated with poor outcome in LSCC (log-rank* P* = 0.024). A multivariate Cox proportional hazard analysis was then performed, by adjusting for age, smoking behavior, histological differentiation, clinical stage, and lymphatic metastasis (Table 3), and the results confirm a significantly poor prognosis in LSCC patients with* SHISA3* methylation (hazard ratio (HR), 2.71; 95% confidence interval, 1.024–7.177;* P* = 0.047).

## 4. Discussion

In cancer research, identification of robust biomarkers, which enable early detection and reliable prognosis of malignancy, is a top priority. DNA methylation is one of the most widely studied epigenetic changes [19]. Previous studies have shown that the inactivation of tumor suppressor genes (TSGs) in numerous cancers, including LSCC, may be attributed to the hypermethylation of CpG islands in the promoter region [20, 21]. DNA methylation can provide an alternative target in the search for biomarkers that could provide a sensitive, specific, and early marker for cancer diagnosis and prognosis [22, 23].* SHISA3* is a newly found TSG in non-small-cell lung cancer, which conducts its tumor suppression activity through the Wnt signaling pathway [15].

In the current study, the methylation status of the* SHISA3* promoter in 93 paired LSCC tissue samples was measured using MSP, revealing that the* SHISA3 *promoter is highly methylated in LSCC. Interestingly, the results indicate that* SHISA3* promoter methylation is more frequently observed in poorly differentiated, lymph node metastasis, and advanced clinical stages, as well as advanced tumor invasion LSCC patients. It can be concluded that* SHISA3* methylation is associated with LSCC clinicopathological characteristics. Additionally, the qRT-PCR results show that the* SHISA3 *expression level is significantly lower in LSCC tissues than in corresponding normal tissue. Methylated* SHISA3 *in LSCC was also associated with a significant reduction in* SHISA3* transcriptional activity, when compared with unmethylated* SHISA3*. The reporter gene activity assay revealed that the* SHISA3* promoter fragment analyzed by MSP was able to regulate gene expression, which implied that the dysregulation of* SHISA3* may attribute to the hypermethylation of promoter region. All these phenomena may be explained by the fact that the SHISA3 protein can protect from malignant transformation and invasion during early stage disease [15], and the dysregulation of* SHISA3 *in LSCC, at least partly, results from methylation [16].

The diagnostic power of methylation biomarkers has been well illustrated in many human cancers [24, 25]. In the present study, the summary ROC curve and AUC were applied to determine the diagnostic value of* SHISA3* methylation for LSCC [26]. When the AUC is close to 1.0, this signifies a good risk predictor [27]. In this study, the sensitivity, specificity and AUC for* SHISA3* methylation in LSCC is 0.99, 0.83 and 0.91, respectively, suggesting that detecting* SHISA3* methylation has a good diagnostic accuracy and represents a potential diagnostic biomarker for LSCC. Additionally, accumulating evidences have shown that the combination of several epigenetic biomarkers can improve the sensitivity and specificity of diagnostic testing for cancers [28, 29]. Therefore, it would be logical to combine* SHISA3 *methylation testing with other methylation biomarkers. This approach requires further studies to determine its potential diagnostic power for LSCC. It is important to point out that according to these findings,* SHISA3* methylation is strongly associated with inferior survival outcomes, since the log-rank test analysis showed that the OS in LSCC patients with hypermethylated* SHISA3* was statistically lower than in those without methylated* SHISA3*. Considering the contribution of a variety of factors (such as age, smoking behavior, histological differentiation, clinical stage and lymphatic metastasis, and* SHISA*3 methylation) to OS, a multivariate Cox proportional hazard analysis was performed to adjust for these factors, and the results confirmed that methylation of* SHISA3* could be an independent unfavorable prognostic factor for LSCC. These results are also supported by previous reports in lung cancer and colorectal cancer [15, 16].

In conclusion, this study has revealed that* SHISA3* promoter hypermethylation is a common event in LSCC, contributing to its transcriptional inactivation, and may be involved in the invasion, progression, and metastasis of LSCC. These findings strongly indicate that methylated* SHISA3* is a new potential epigenetic biomarker for the early diagnosis and prognosis of LSCC. However, further research is needed to elucidate the underlying mechanisms of the* SHISA3* gene in the pathogenesis of LSCC.

## Figures and Tables

**Table 1:** List of all primers used for MSP and qRT-PCR assay.

Primer	Sequence (5′ to 3′)	Amplification size (bp)
Methylation		204
Forward	AGAGGTGATCGGTAATTTTTTAGTC	
Reverse	CCTATTACACAAACTCAAACTCGTT	
Unmethylation		203
Forward	GAGGTGATTGGTAATTTTTTAGTTG	
Reverse	CCTATTACACAAACTCAAACTCATT	
*SHISA3* qRT-PCR		85
Forward	GTCTACGTCCCCTTTCTCATCG	
Reverse	AGGTGCAACAATAAATAGCCACT	
*GAPDH* qRT-PCR		
Forward	CCATGGAGAAGGCTGGGG	194
Reverse	CAAAGTTGTCATGGATGACC	

**Table 2:** Association between the *SHISA3* methylation in LSCC and the clinicopathological features.* N*: number; M: methylation; U: unmethylation; ^#^*P* value calculated by Fisher's exact test.

Characteristics	*SHISA3* methylation				
	*N*	M	U	*χ* ^2^	*P*
Gender					
Female	4	3	1	—	1^#^
Male	89	63	26		
Age					
<60	49	36	13	0.31	0.57
≥60	44	30	14		
Smoking behavior					
Yes	75	53	21	0.08	0.78
No	18	13	6		
Histological grade					
Well/moderately	80	53	27	—	0.02^#^
Poorly	13	13	0		
Clinical stage					
I + II	48	29	19	5.36	0.02
III + IV	45	37	8		
Tumor invasion					
1 + 2	58	37	21	3.85	0.05
3 + 4	35	29	6		
Lymph node metastasis					
No	64	41	23	4.75	0.03
Yes	29	25	4		

**Table 3:** Multivariate Cox proportional hazards analysis in 93 LSCC patients.* N*: number; Ref: reference category; HR: hazard ratio; CI: confidence interval.

Characteristics	*N*	*P *value	HR	95% CI
Age	93	0.979	0.999	0.953–1.048
Smoking behavior				
No (Ref)	18	—	1	—
Yes	75	0.288	0.636	0.276–1.465
Differentiation				
Well (Ref)	45	—	1	—
Moderated	35	0.256	0.656	0.318–1.356
Poorly	13	0.24	0.512	0.167–1.566
Clinical stage				
Stage I (Ref)	29	—	1	—
Stage II	19	0.783	1.196	0.336–4.257
Stage III	12	0.053	3.735	0.982–14.208
Stage IV	33	0.019	4.238	1.272–14.117
Lymphatic metastasis				
No (Ref)	64	—	1	—
Yes	29	0.723	1.172	0.489–2.809
Methylation				
No (Ref)	27	—	1	—
Yes	66	0.045	2.71	1.024–7.177
